# TRAPP webserver: predicting protein binding site flexibility and detecting transient binding pockets

**DOI:** 10.1093/nar/gkx277

**Published:** 2017-04-20

**Authors:** Antonia Stank, Daria B. Kokh, Max Horn, Elena Sizikova, Rebecca Neil, Joanna Panecka, Stefan Richter, Rebecca C. Wade

**Affiliations:** 1 Molecular and Cellular Modeling group, Heidelberg Institute for Theoretical Studies (HITS), Heidelberg, Baden-Württemberg 69118, Germany; 2 Heidelberg Graduate School of Mathematical and Computational Methods for the Sciences, Heidelberg University, Heidelberg, Baden-Württemberg 69120, Germany; 3 Interdisciplinary Center for Scientific Computing (IWR), Heidelberg University, Heidelberg, Baden-Württemberg 69120, Germany; 4 Center for Molecular Biology of the University of Heidelberg (ZMBH), DKFZ-ZMBH Alliance, Heidelberg University, Heidelberg, Baden-Württemberg 69120, Germany

## Abstract

The TRAnsient Pockets in Proteins (TRAPP) webserver provides an automated workflow that allows users to explore the dynamics of a protein binding site and to detect pockets or sub-pockets that may transiently open due to protein internal motion. These transient or cryptic sub-pockets may be of interest in the design and optimization of small molecular inhibitors for a protein target of interest. The TRAPP workflow consists of the following three modules: (i) TRAPP structure— generation of an ensemble of structures using one or more of four possible molecular simulation methods; (ii) TRAPP analysis—superposition and clustering of the binding site conformations either in an ensemble of structures generated in step (i) or in PDB structures or trajectories uploaded by the user; and (iii) TRAPP pocket—detection, analysis, and visualization of the binding pocket dynamics and characteristics, such as volume, solvent-exposed area or properties of surrounding residues. A standard sequence conservation score per residue or a differential score per residue, for comparing on- and off-targets, can be calculated and displayed on the binding pocket for an uploaded multiple sequence alignment file, and known protein sequence annotations can be displayed simultaneously. The TRAPP webserver is freely available at http://trapp.h-its.org.

## INTRODUCTION

Protein flexibility plays a key role in molecular recognition but is often neglected in protein structure-based drug design projects. Thus, transient or cryptic pockets that are not visible in available protein crystal structures but may bind ligands are missed. Computational approaches to identify transient binding pockets or sub-pockets provide a means to reveal druggable pockets and to expand the possibilities for improving the specificity and diversity of designed compounds. Indeed, consideration of protein binding pocket dynamics has played an important role in drug discovery (1). For example, consideration of pocket dynamics in p38 mitogen-activated protein kinase helped to find an inhibitor (2). Another example is the identification of a cryptic pocket in HIV integrase, adjacent to the known active site, in molecular dynamics (MD) simulations (3). This pocket was exploited in the discovery of HIV integrase inhibitors, leading to the development of the drug Raltegravir (4). MD simulations have also revealed a transient and potentially druggable binding pocket at the dimeric interface of HIV-1 protease (5).

Most often, ligand design is undertaken for known binding sites rather than novel sites. The known binding sites may be sites where natural ligands bind or where existent drugs (or active compounds) bind. Therefore, we have designed the TRAPP webserver as a tool for studying the dynamics of a known binding pocket or any other protein cavity of interest, and for identifying and characterizing transient sub-pockets. These transient sub-pockets may be considered for ligand design and optimization, and therefore the TRAPP webserver provides information on their physicochemical and sequence properties, as well as shape and dynamics.

A range of computational tools for detecting binding pockets on protein structures and analyzing their static structures is available (see https://bioinformatictools.wordpress.com/tag/pocket-finder/ and the recent reviews by Zheng *et al*. (6) and Krone *et al*. (7)). Several of them, trj_cavity (8), MDpocket (9) based on FPocket (10,11), EPOCK (12), PocketAnalyzer^PCA^ (13), POVME 2.0 (14), EPOS^BP^ (15), as well as the previous version of TRAPP web server (16), are designed for analysis of binding pocket dynamics in MD simulation trajectories. Some of them also provide pocket characteristics, such as the volume or hydrophobicity, and analysis of changes in pocket shape (as defined by pocket occupancy (14), principle component analysis of the pocket motion (13) or clustering of pocket regions (15)). They all, however, require the user to provide and, in some cases to align and superimpose, snapshots of the trajectory to be analyzed. The trajectory is usually a standard MD trajectory, but these are typically too short to sample all the cryptic pockets that are potentially interesting for drug design. Indeed, protein binding pocket dynamics are dependent on a wide variety of motions ranging from small side-chain vibrations and rotations to large-scale changes in secondary structure and domain movements. Complete sampling of such motions is difficult to achieve in standard MD simulations. On the other hand, several approaches have been developed that enable protein flexibility to be explored in a relatively short simulation time (17,18). In particular, it has been demonstrated that the opening of cryptic pockets appearing on the microsecond time scale can be revealed in several-nanosecond L-RIP simulations (18). These simulations, though less accurate than standard MD, give the user insight into the transient hot spots of the binding pocket. Thus, the main motivation of the present development of the TRAPP webserver was to provide the user with an automated workflow to invoke a toolbox of methods to explore pocket flexibility arising from protein conformational changes on a wide range of temporal and spatial scales. The current TRAPP webserver provides a choice of several methods to efficiently generate conformational ensembles representing binding pocket dynamics, in addition to the option of uploading conformational ensembles, e.g., crystal structures or MD trajectories, generated by other tools. Furthermore, the integrated alignment procedure makes it possible to compare binding pockets in proteins from different species, e.g. with gaps or insertions, or with mutated residues. Additionally, the TRAPP webserver provides new tools to analyse the geometric, dynamic and physicochemical properties of the transient pockets, along with protein sequence conservation and differential conservation between on-targets and an off-target. The present TRAPP webserver also allows simultaneous visualization of residue-based mutations by integration of queries with the ProSAT^+^ webserver (19). Thus, the TRAPP webserver provides capabilities for a wide-ranging interactive analysis of pocket dynamics for transient sub-pocket detection and characterization on the basis of a single input protein structure. We here describe the structure and implementation of the TRAPP webserver, the user input, and the results produced. We illustrate the use of the TRAPP webserver by means of an example application to a protein target for anti-parasitic drug design.

## MATERIALS AND METHODS

**Figure 1.**
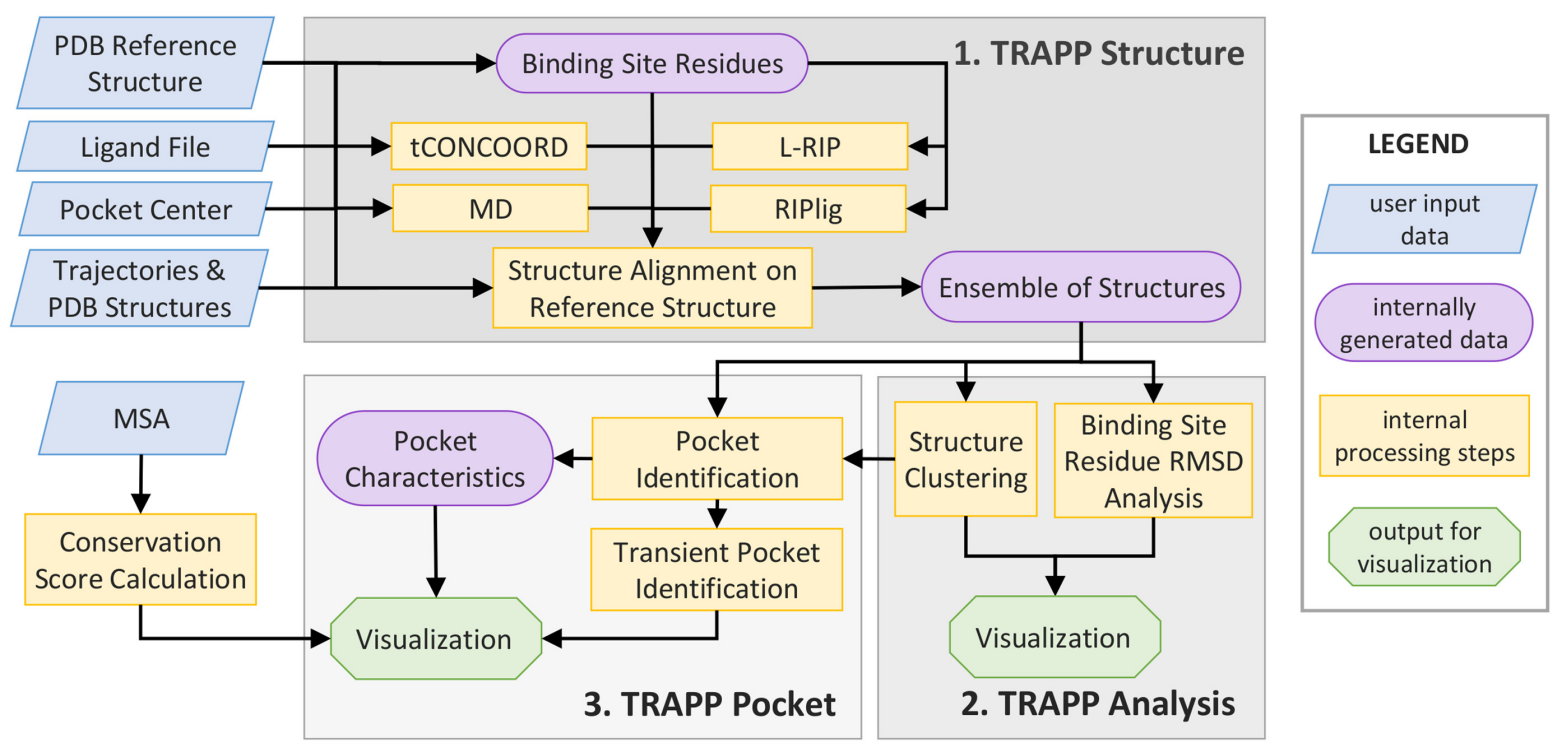
Workflow of the TRAPP webserver.

The overall workflow of the TRAPP webserver consists of three modules, as illustrated in Figure 1: (i) *TRAPP Structure*, (ii) *TRAPP Analysis*, and (iii) *TRAPP Pocket*. For each of these steps, the user can select and define additional simulation parameters in the user interface. After an initial user input, *TRAPP Structure* and TRAPP *Analysis* are started sequentially. The *TRAPP Pocket* module is then run after further user input.

Here, we provide a short description of the methods used and the simulation results provided. A detailed documentation of each module, input parameters and output data, as well as several usage examples, is available on the TRAPP web server.

### Input

Initially, the user is required to upload a reference protein structure in PDB format, and define the center of the binding pocket of interest. The latter is specified either by uploading a PDB file containing the coordinates of a ligand, or by manually defining the coordinates of the center of the pocket together with the distance within which protein residues are considered as *binding site residues*. They are used later for structure alignment and superposition. Further, the user can select one or several simulation methods to be used in the *TRAPP Structure* module for exploring protein conformations, or upload trajectories or PDB files. Optionally, the user may provide a multiple sequence alignment (MSA) file in FASTA format to analyse the sequence conservation of the binding pocket.

### Functionality and output of the workflow modules

#### TRAPP structure

The aim of this module is to enable fast generation of ensembles of protein structures that represent the conformational diversity of the binding pocket. The user can choose to use one or more of the following methods to generate an ensemble of structures: short implicit solvent MD simulation (20), tCONCOORD (17), L-RIP and RIPlig (18). All the methods are described in detail in the respective publications. The short MD simulations mainly allow for the sampling of side chain movements, whereas the MD-based perturbation approaches, L-RIP and RIPlig, are useful for sampling larger scale motions of the binding pocket, including backbone motions, domain motions and changes of secondary structure elements. tCONCOORD provides a constraint-based sampling methodology for side-chain and loop motions, but can also be useful for exploring slow domain movements (21). The generated trajectories or ensembles of snapshots are directly transferred to the *TRAPP Analysis* module for clustering and analysis of protein flexibility.

#### TRAPP analysis

This module provides tools for the comparison of the binding pockets in the protein structures uploaded or generated in the previous step, *TRAPP Structure*. To this end, all structures are aligned and superimposed using the backbone atoms of the binding site. For clustering of the binding site conformations, one of two different metrics can be selected: RMSD of backbone atoms (default) or RMSD of geometric center of the non-hydrogen atoms of the binding site residues. By default, a simplified single-linkage hierarchical clustering algorithm (16) is used with a RMSD threshold of 3 Å. The clustering procedure can be refined by using a smaller threshold and/or k-means clustering (16). The set of binding site residues can be increased or reduced at this step. A binding site residue RMSD matrix for all structures, the RMSD averaged over all binding site residues for each structure, and the maximum RMSD of each binding site residue in all structures are calculated and plotted. All three-dimensional structures of the cluster representatives are displayed together with the reference structure in a JSmol applet.

#### TRAPP pocket

The *TRAPP pocket* module tracks pockets in the protein structures and identifies transient regions. Protein cavities located at the binding site are calculated for each structure as described in (16) and stored on a grid. Additionally, the physicochemical properties of the side chains that directly contact the binding cavities (e.g. positive/negative charge, H-bond donors/acceptors), pocket volume, and surface area are calculated and displayed. Furthermore, the pocket lining residues are analyzed for all structures and compared graphically to those in the reference structure. The pocket dynamics are analysed, and transient regions (those that either appear or disappear relative to the reference structure provided as an input by user) and conserved regions (defined by the percentage presence in snapshots or structures) are identified. Appearing and disappearing regions are displayed by isocontours in JSmol along with plots showing in which structure or snapshot they occur. Transient pocket regions occurring in 25% and 50% (default values that can be altered by the user) of the structures are split into compact sub-regions and displayed. The extent of the opening of each of the transient sub-pockets is computed for each snapshot and plotted.

If the user uploads a MSA, a conservation score is calculated per residue using a python-based tool (22) that applies the Jensen–Shannon divergence score. This allows a quantification of the similarity between probability distributions, by taking a background amino acid distribution (BLOSUM62) into account. This score is rescaled to the range between 30 and 70, mapped on the protein structure, and displayed by color (blue: low conservation, red: high conservation) together with the *TRAPP Pocket* results. Another option allows the user to upload a MSA file containing a set of protein sequences representing an on-target group and one sequence representing an off-target. The conservation score is calculated twice: once with the MSA containing the off-target sequence, and once without it. Afterwards, the absolute difference of these two conservation scores per residue is calculated, rescaled, and mapped on the three-dimensional structure. In this case, blue highlights residues with high similarity between on- and off-target sequences and red indicates residues with low similarity. The MSA is displayed with MSAviewer (23) and the sequence conservation can be shown by colour coding the reference structure. The TRAPP webserver also provides a coupled visualization of residue-based annotations in ProSAT^+^.

All data and graphics generated by the *TRAPP Analysis* and *TRAPP Pocket* modules can be downloaded as raw data or as a compressed archive. Additionally, a PyMOL session is generated and can be downloaded with other data as a single archive for in-depth visual inspection of the protein structures and pocket analyses.

### Technical design of the TRAPP webserver

The TRAPP webserver is implemented in Java and uses the Play framework 2.5. For the HTML front-end, the Twitter Bootstrap (version 3.3.7) is used to provide a clear layout of the user interface. All visualizations of the three-dimensional structures and pocket information employ the Java Script-based JSmol (https://sourceforge.net/projects/jsmol/). This enables a Java-free visualization in most common web browsers, which has the advantage that no Java installation is required on the client site and associated security issues can be avoided. The TRAPP modules are implemented in Python and Fortran (16). After each submission of a TRAPP job, the user is provided with a session identifier link, which can optionally be sent by email. This session link allows the user to monitor the status of their current job and access their results. This session link, which is only provided to the user who started the analysis, also allows the user to share their results with other people. The runtime of the submitted jobs depends on which method is selected in the *TRAPP structure* module and can vary between a few minutes and several hours. Submitted jobs are automatically distributed on a compute cluster. The user can select to receive a notification of job completion by email. The TRAPP results are stored for a limited period during which they can be accessed and downloaded.

## RESULTS

We illustrate the capabilities of the TRAPP webserver by means of the following example application case. Additional examples can be found on the TRAPP webserver. Examples of the application of the TRAPP procedure, and of MD simulations and the L-RIP and RIPlig methodologies to explore protein pocket dynamics, can be found in (15,18).

### Example application case

Dihydrofolate reductase (DHFR) is a key enzyme in the folate pathway, and therefore in DNA synthesis. It is a known target for anti-cancer antifolate drugs, but antifolates are also being investigated as potential drugs against human trypanosomatid parasites (24). However, the development of selective anti-parasitic antifolates without side effects is hindered by the fact that the binding pocket of trypanosomatid DHFR is very similar to that of human DHFR (hDHFR). Therefore, it is essential to identify DHFR binding pocket features that could differentiate parasite variants (on-targets) from the human one (off-target). Here, we provide an example of how this problem can be addressed by analysis of the binding site flexibility with the TRAPP webserver.

There are several crystal structures available for the DHFR of *Trypanosoma cruzi*, a human parasite causing Chagas’ disease. Thus, we analyse the conformational variability of the binding site of *T. cruzi* DHFR (TcDHFR), and compare its binding site sequence with that of the off-target protein, hDHFR. We include DHFR sequences of other representative trypanosomatid species (other potential on-targets) in the alignment to identify amino acid residues conserved in trypanosomatids.

The 9 crystal structures available for TcDHFR are (resolution and number of chains in parentheses): 2h2q (2.4 Å, 2), 3cl9 (3.3 Å, 1), 3clb (3.0 Å, 4), 3hbb (3.0 Å, 4), 3kjs (2.5 Å, 4), 3inv (2.4 Å, 2), 3irm (2.1 Å, 4), 3irn (2.6 Å, 4), 3iro (2.8 Å, 4). To prepare these structures for TRAPP, we split the PDB files into 29 files, each containing a single protein chain with only ATOM records retained. (We discard chain B of 2h2q because of many missing residues around the binding site.) We assign PDB file 3cl9 as the reference structure, extract the bound ligand, methotrexate, into a separate ligand file in PDB format, and use it to define the binding site (5 Å radius). To examine the on/off-target selectivity, we generate a multiple sequence alignment (MSA) in FASTA format for five trypanosomatid DHFR sequences, to represent the on-target group (UniProt: Q27793, Q27783, P07382, Q8MQV3, E9B8U9), and for the off-target hDHFR (UniProt: P00374).

**Figure 2.**
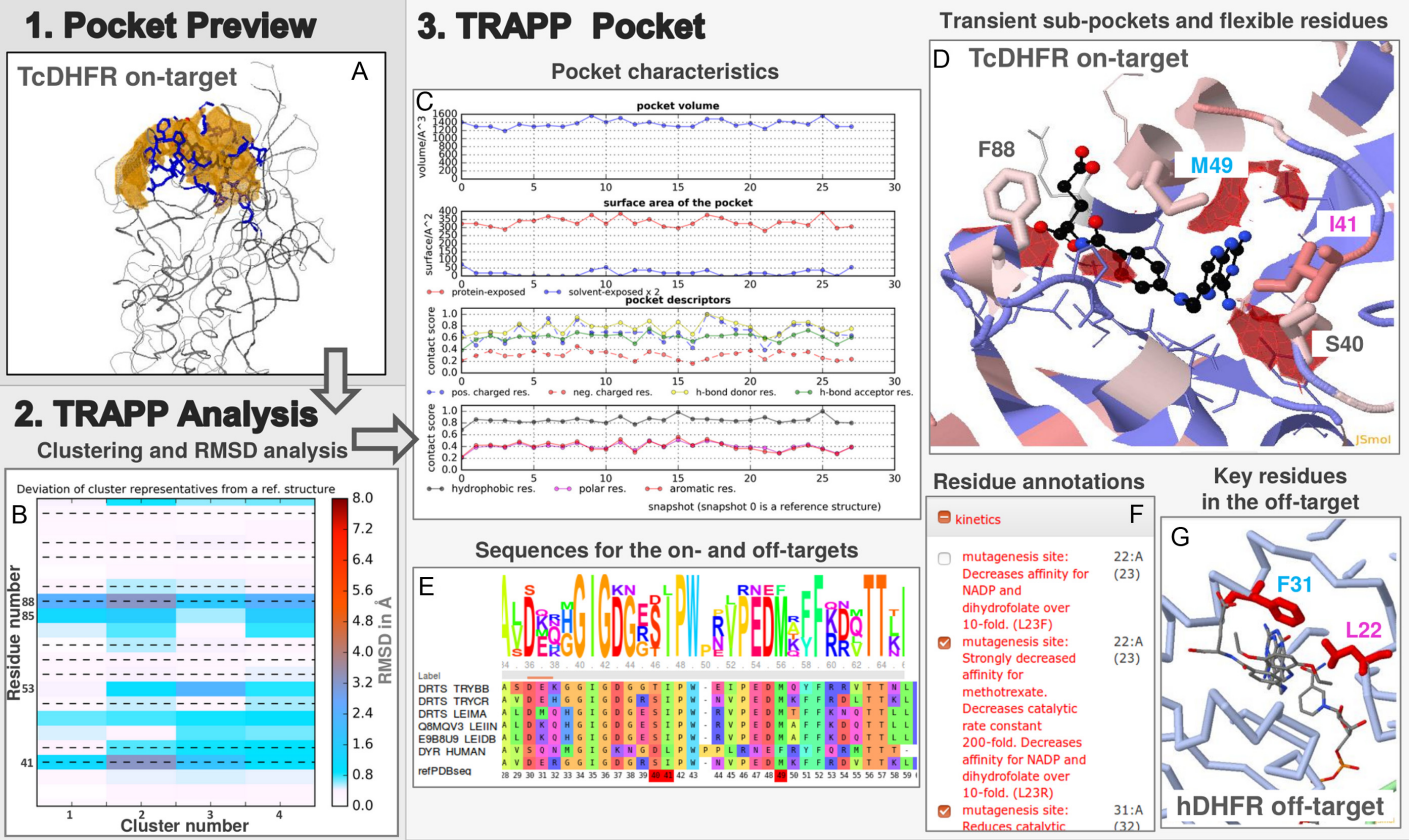
Screenshot images from an example application of the TRAPP webserver to the analysis of the binding site of a parasite enzyme to identify transient sub-pockets that are selective with respect to the human homologue (see text for details): (**A**) the binding pocket in the TcDHFR reference structure is shown by orange isocontours; (**B**) the clustering of structures performed by the *TRAPP Analysis* module is illustrated by the RMSD of the binding site residues from the reference structure for four cluster representatives; (**C**) the pocket characteristics generated by the *TRAPP Pocket* module are shown for all analyzed structures; (**D**) sub-pockets detected in crystal structures but absent in the reference structure are shown by red isocontours, residues that are not conserved between the on- and off-targets (TcDHFR and hDHFR) are labelled; (**E**) multiple sequence alignment showing the binding site sequence conservation; (**F** and **G**) illustration of key residues identified: residues I41 and M49 in TcDHFR (D) correspond to L22 and F31 in hDHFR (G), whose mutation is known to affect inhibitor binding and catalytic activity in hDHFR (F).

With these input data prepared, we start TRAPP *Analysis*. Screenshot images that illustrate the analysis and results are shown in Figure 2, (further screenshots and instructions for this example are available on the website of the TRAPP webserver). After uploading the prepared data into the user interface, we inspect a summary of all parameters, a preview of the pocket in JSmol (Figure 2A), and a list of all defined binding site residues. The TRAPP *Analysis* results show the RMSD values of the binding site residues for each uploaded structure to those in the reference structure. In addition, the RMSD values for the four cluster representatives (*k*-means, 1.5 Å threshold using geometric centers of side-chains) are shown together in one plot (Figure 2B). This analysis reveals the greatest structural variability at the binding site residues Ile41, Arg53, Pro85 and Phe88.

Next, we run TRAPP *Pocket* with the default parameter values. In the summary of the results, we select a checkbox to color the reference structure according to the per-residue differential sequence conservation (blue—conserved, red—not conserved between the on-targets and off-target). In the dropdown list for appearing pockets, we select the threshold of 50% to show in the JSmol applet structure view red isocontours for the regions that appear in 50% of all the uploaded structures compared to the reference structure. We observe opening of several small sub-pockets near Ser40/Ile41, Met49 and Phe88 (Figure 2D). The displayed MSA (Figure 2e) shows full conservation of amino acid positions 41, 49 and 88, and partial conservation of position 40, between the representative trypanosomatids. The coloring of the 3D structure shows that these positions differ from the corresponding residues in hDHFR (Figure 2D). Notably, these residues are characterized by differing size and/or charge in parasite vs. human DHFR (Met49 versus Phe, Phe88 versus Asn, and Ser/Thr40 versus Asp). The integrated ProSAT^+^ tool further shows that the residues identified as flexible, non-conserved, and close to an appearing sub-pocket, Ile41 (Leu22 in hDHFR, both labeled in magenta in Figure 2D and G) and Met49 (Phe31 in hDHFR, both labelled in cyan in Figure 2D and G), are known mutagenesis sites in hDHFR (Leu22 to Arg, and Phe31 to Arg, Figure 2F) that affect the binding affinity of the anti-cancer drug, methotrexate (25), or the catalytic activity (26).

In summary, this quick analysis with the TRAPP webserver highlights transient pocket regions and residues in the structure that can provide starting points for the design of selective drugs targeting parasite DHFR but not human DHFR. In fact, Schormann *et al*. could design an inhibitor about 7 times more active (in terms of K_i_ value) against TcDHFR than hDHFR by targeting the region close to Met49 (27).

## DISCUSSION

The TRAnsient Pockets in Proteins (TRAPP) webserver is a platform to facilitate the exploration of the dynamics of a protein binding site, including variations in pocket physicochemical properties, and the detection of transient pockets and sub-pockets. Conformational variations of the binding site may arise due to protein internal flexibility ranging from side-chain rotations to large-scale conformational changes in the secondary and tertiary structure. For this purpose, an ensemble of different experimental or simulated protein structures is analysed, which can either be provided by the user or be generated by using several provided methods. Additionally, the webserver provides an analysis of the binding site residue conservation and known sequence annotations in the context of transient binding pocket analysis results.

The TRAPP webserver has a modular structure. It currently provides four methods to generate conformational ensembles and is designed so that further methods can be added in the future. A challenge for the web server design is the fact that some of these methods are computationally demanding, meaning that, despite parallelization, job run times prevent uninterrupted interactive use of the TRAPP webserver for an analysis. Future improvements in the computing abilities of the back-end, increases in compute cluster size, or linking of the TRAPP webserver to cloud computing resources, will increase the capacity and speed of the TRAPP webserver and ameliorate these issues. The modular design of the TRAPP webserver also permits the addition of further pocket analysis features, e.g. a sub-pocket specific druggability score or a pocket-ligand similarity score. Both features would provide additional quantitative data to aid the design of more specific compounds that take advantage of certain sub-pockets and their biochemical features.

The TRAPP webserver enables the analysis of the structural plasticity of a binding pocket, and the identification of transient pocket regions and residues important for selectively targeting a specific protein. Together with the sequence-based conservation analysis of on-target and off-target groups, it enables the identification of residues that distinguish the two groups, providing a basis for developing more selective drugs.
